# The mixture of *Agrimonia pilosa* Ledeb. and *Salvia miltiorrhiza* Bunge. extract produces analgesic and anti-inflammatory effects in a collagen-induced arthritis mouse model

**DOI:** 10.1080/19768354.2022.2106302

**Published:** 2022-08-08

**Authors:** Jing Hui Feng, Jeon Sub Jung, Seung Hwan Hwang, Soo Kyeong Lee, Sang Youn Lee, Youn Gil Kwak, Doo-Ho Kim, Chu-Youn Song, Min Jung Kim, Hong Won Suh, Sung Chan Kim, Soon Sung Lim

**Affiliations:** a Department of Pharmacology, College of Medicine, Hallym University, Chuncheon, Republic of Korea; b Institute of Natural Medicine, College of Medicine, Hallym University, Chuncheon, Republic of Korea; c Department of Food Science and Nutrition, College of Natural Science, Hallym University, Chuncheon, Republic of Korea; d R&D Center, Huons Co., Ltd., Ansan, Republic of Korea; e Research Institute, Huons Foodience, Keumsan, Republic of Korea; f Department of Biochemistry, College of Medicine, Hallym University, Chuncheon, Republic of Korea; g Institute of Korean Nutrition, Hallym University, Chuncheon, Republic of Korea

**Keywords:** *Agrimonia pilosa* Ledeb, *Salvia miltiorrhiza* Bunge, mixture extract, antinociception, arthritis

## Abstract

Pain and inflammation typically manifest in patients with arthritis. It is now widely known that *Agrimonia pilosa* Ledeb (AP) and *Salvia miltiorrhiza* Bunge (SM) exert anti-inflammatory and antinociceptive effects. We have previously reported that the mixture extract (ME) from AP and SM produces antinociceptive and anti-inflammatory effects in gout arthritis and monoiodoacetate (MIA)-induced arthritis models. In the present study, we assessed the antinociceptive and anti-inflammatory effects on the collagen-induced arthritis (CIA) model. The antinociceptive effects in mice were measured using the von Frey test. ME administered once or for one week (once per day) once, and one-week reduced the pain in a dose-dependent manner (from 50 to 100 mg/kg) in the CIA-induced osteoarthritis (OA) model. ME treatment also reduced tumor necrosis factor (TNF)-α and C-reactive protein (CRP) levels in plasma and ankle tissues. Furthermore, COX-1, COX-2, NF-κB, TNF-α, and IL-6 expressions were attenuated after ME treatment. In most experiments, the antinociceptive and anti-inflammatory effects induced by ME treatment were almost equal to or slightly better than those induced by *Perna canaliculus* (PC) treatment, which was used as a positive control. Our results suggest that ME possesses antinociceptive and anti-inflammatory effects, indicating its potential as a therapeutic agent for arthritis treatment.

## Introduction

Arthritis is a painful disease of the joints that affects the lifestyles of millions of patients (Wieland et al. 2005). Arthritis is generally caused by mechanical damage to joints, cartilage matrix composition, and structural changes caused by aging (Goldring and Goldring 2007). Nonsteroidal anti-inflammatory drugs (NSAIDs) or opioids are usually prescribed to patients with arthritis. However, NSAIDs and opioids have side effects such as peptic ulcers, constipation, and dizziness (Bijlsma et al. 2011). Therefore, a new and effective therapeutic strategy for arthritic patients is needed.

Several lines of evidence have demonstrated that *Agrimonia pilosa* Ledeb (AP), a traditional herb, exhibits anti-inflammatory effects (Jang et al. 2017; Kim et al. 2020). *Salvia miltiorrhiza* Bunge (SM) also exhibits anti-inflammatory effects (Jeon et al. 2008; Gao et al. 2016; Luo et al. 2019). We have previously reported that the crude extract of AP induces antinociceptive effects in several pain models, such as the tail-flick test and acetic acid-, formalin-, and monosodium urate (MSU)-induced pain models (Feng et al., 2021; Park et al. 2012). Furthermore, tanshinones, a component of SM, exhibited antinociceptive effects in a rat neuropathic pain model (Mannelli et al. 2018; Zhang et al. 2019). These findings led us to speculate that the mixture extracts (ME) from AP and SM would produce antinociceptive and anti-inflammatory effects in a collagen-induced arthritis (CIA) model.

Several studies have reported that C-reactive protein (CRP), leukotriene B4 (LTB_4_), prostaglandin E2 (PGE_2_), interleukin 6 (IL-6), and tumor necrosis factor-alpha (TNF-α) play crucial roles in inflammatory processes and the pathophysiology of arthritis (Choy and Panayi 2001; McInnes and Schett 2007; Abramson 2008; Goldring and Otero 2011; Ahn et al. 2021). It has been suggested that the reduction of such inflammatory cytokine levels can be an effective method of curing arthritis (Libby 2008). Both AP and SM have been found to inhibit the release of inflammatory cytokines *in vitro* (Chen et al. 2016; Choi et al. 2018; Ngo et al. 2021; Kim et al. 2020). Thus, in the present study, we characterized the mechanism of ME in inflammation and arthritis pain *in vivo* and *in vitro*.

Several studies have reported that the extracts from *Perna canaliculus* (PC) show a strong anti-inflammatory effect, suggesting that PC can be used as an herbal therapeutic for the treatment of arthritis (Eason et al. 2019; Siriarchavatana et al. 2019). Thus, in the present study, we compared the antinociceptive and anti-inflammatory effects of ME and PC in a CIA model.

## Methods and materials

### Material preparation

Dried AP (Leaf) and SM (Root) were purchased from a local market in Yeongcheon, Gyeongsangbuk-do Province, Korea (June 2019), and were ground to granule size before extraction. A mixture of powdered AP (80 kg) and SM (20 kg) was extracted in 50% EtOH (1,200 L × 2 times) at 70°C for 7 h and filtered with a 60-mesh filter. The extract lyophilized and yielded brown powders (25.16 kg, 25.16%). The mixture extract contained 1.24 mg/g of rutin and 28.39 mg/g of salvianolic acid as a specific ingredient, as identified using high-performance liquid chromatography. Stabilized PC powder (Pernatec^TM^, 931200) was purchased from Waitaki Biosciences (Christchurch, New Zealand). The reagents and solvents were purchased from Sigma-Aldrich Co.

### Experimental animals

Male Institute of Cancer Research (ICR) mice (4–6 weeks old, 20–25 g) were purchased from MJ Co., Ltd. (Seoul, Korea). Five mice were housed per cage with food and water *ad libitum* in a room maintained at 22 ± 0.5°C with an alternating 12 h light–dark cycle. The mice were used only once. All animal care and experimental procedures were conducted in accordance with the National Institutes of Health and the ethical guidelines of the International Association for the Study of Pain. The Care and Use of Laboratory Animals protocol was approved by the Animal Care and Use Committee of Hallym University (Registration Number: Hallym 2016-53).

### Production of mouse collagen-induced arthritis (CIA) model

Bovine type II collagen (Chondrex, USA) (2 mg/mL) was prepared in 0.05 M acetic acid buffer, vortexed for 10 min, and mixed with complete Freund’s adjuvant (CFA, Chondrex, USA) in the same ratio (1:1). The solution was dissolved slowly at 4°C for 6 h. Mixed collagen (100 µL) was injected into the tail vein (intradermal injection, i.d.) using a 1 mL gas-tight syringe (Hamilton, 26 gauge). On day 21, after the primary immunization, bovine type II collagen (2 mg/mL) was mixed with incomplete Freund’s adjuvant (IFA; Chondrex, USA) in the same ratio (1:1). The solution was dissolved slowly at 4°C for 6 h. Mixed collagen (100 µL) was injected into the tail vein. Four days after CIA model production, the pain threshold was significantly decreased, and this decreased pain threshold was maintained for up to 28 days. Oral administration of ME or PC (50 or 100 mg/kg, respectively) was initiated 4 days after CIA model production. The von Frey test was used to assess the pain thresholds of the mice. According to our previous procedures (Hong et al. 2020), the mice were allowed to adapt to the test environment for 30 min by placing them in a transparent glass cell with a metal mesh floor. The von Frey filament (North Coast Medical, Inc., Gilroy, CA, USA) was then applied to the plantar surface using the up and down paradigm (Bonin et al. 2014). The hind paw withdrawal thresholds of mice were then compared.

### Measurement of TNF-α and CRP levels in the plasma and ankle tissue in the CIA model

The levels of cytokines, such as TNF-α, CRP, and IL-6, in the plasma or ankle tissues were measured 1 h after oral treatment with ME or PC. Blood (1 mL) was collected by puncturing the retro-orbital venous plexus and centrifuged at 4°C to separate plasma, which was stored at −80°C. The ankle tissues were dissected and homogenized, followed by centrifugation at 4°C. The supernatant was stored at −80°C till further analysis. The level of CRP was evaluated using a mouse CRP ELISA kit (E-EL-M0053, Elabscience Biotechnology Inc.). TNF-α levels were measured using the Mouse TNF-α ELISA kit (MTA00B, R&D Systems). IL-6 levels were measured using a Mouse IL-6 ELISA kit (MTA00B, R&D Systems).

### Western blotting

Proteins were extracted using lysis buffer and quantified using the Bradford method (Bio-Rad Laboratories, Hercules, CA, USA), as described previously (Hong et al. 2020). The samples were separated using sodium dodecyl sulfate-polyacrylamide gel electrophoresis and transferred to polyvinylidene fluoride membranes (Millipore, Bedford, MA, USA). After blocking, the membranes were immunoblotted with primary antibodies COX-1(Abcam, 1:1000), COX-2 (LSBio, 1:1000), NF-κB (Cell Signaling Technology, 1:1000), TNF-α (Abcam, 0.2 μg/ml), or IL-6 (Thermo Fisher, 1:1000), and β-actin (Cell Signaling Technology, 1:1000) at 4°C overnight. After washing, the membranes were incubated at 37°C for 1 h with the horseradish peroxidase (HRP)-conjugated secondary antibodies (Enzo Life Sciences, 1:4000). An enhanced chemiluminescence reagent (Millipore, Billerica, MA, USA) was added, and the light emission was detected using a luminescent image analyzer (LAS-4000, Fuji Film Co., Japan). The intensity of bands was measured using Multi-Gauge Version 3.1 (Fuji Film Co., Japan). The value of each sample was expressed as the percentage of the control tested protein relative to the internal reference, β-actin.

### Statistical analysis

**Figure 1.**
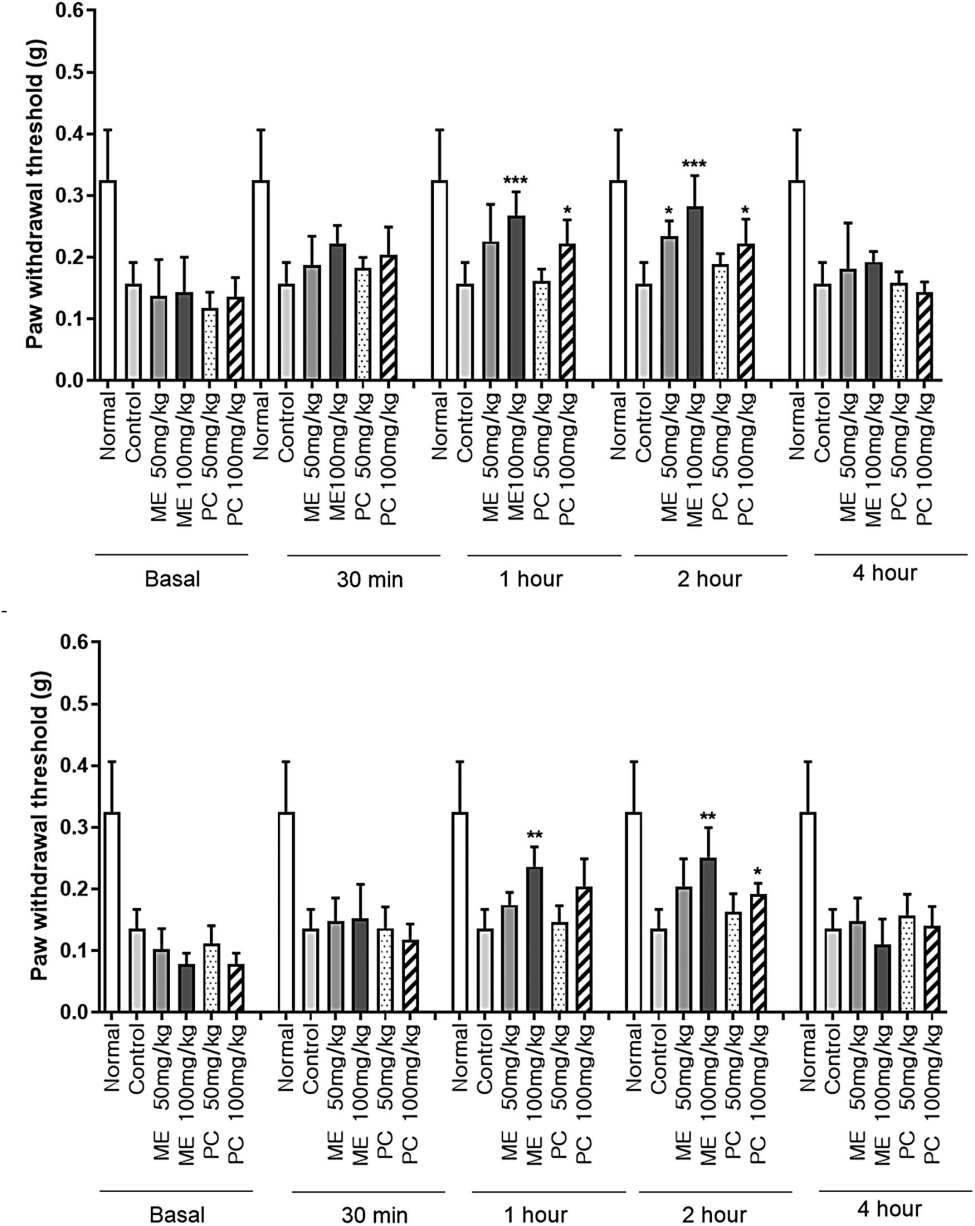
The effect of mixture extract (ME) and *Perna canaliculus* (PC) administration on pain threshold in the collagen-induced arthritis (CIA) model. Mice were orally administered with the vehicle, ME (50 or 100 mg/kg), or PC (50 or 100 mg/kg), and the pain threshold was measured 30, 60, 120, and 240 min after treatment using the von-Frey test. (A) Mice were treated with the vehicle, ME, or PC once. (B) Mice were repeatedly administered (once per day for 1 week) orally with the vehicle, ME, or PC treatments. The values denote the mean ± SD. The number of animals used for each group was 5 (*p < 0.05, **p < 0.01, ***p < 0.001, compared to the vehicle-treated normal group; ^+^*p* < 0.05, ^++^*p* < 0.01, compared to the CIA control group).

All values are expressed as mean ± standard deviation (SD). GraphPad Prism (Version 8.4.2, GraphPad Software, USA) was used for statistical analysis. As shown in Figure 1, multiple groups were compared at several time points using repeated measures ANOVA followed by Tukey’s post-hoc test. As shown in Figure 3, data were compared and analyzed using two-way ANOVA with Tukey’s post-hoc test. The unpaired *t*-test was used to assess the differences between the two groups. Statistical significance was set at *P <* 0.05.

## Results

### Effect of ME and PC on pain threshold in CIA model

Four days after CIA model development, 50 or 100 mg/kg of ME and PC were orally administered once or repeatedly (once per day for 1 week). As shown in Figure 1, both the single and repeated treatments with ME caused the reversal of the decreased pain threshold, as revealed in the CIA model. The antinociceptive effects of ME were observed at both 50 and 100 mg/kg. The antinociceptive effect was observed 30 min after the single ME administration; the paw withdrawal thresholds remained higher than those of the control treatment 2 h after ME administration, and the antinociceptive effect of ME returned to that of the control treatment 4 h after ME administration (Figure 1(A)). Additionally, ME treatment showed antinociception from 1 to 2 h, even after repeated administration (Figure 1(B)). Furthermore, ME-induced antinociceptive effects were more pronounced than PC-induced effects in the CIA model. The onset time of antinociception in the group where ME was administered once or for one week was faster than that in the group where PC was administered once or for one week (Figure 1(A,B)).

### ME and PC suppress the levels of CRP and TNF-α levels in the plasma and ankle tissue in the CIA model

**Figure 2.**
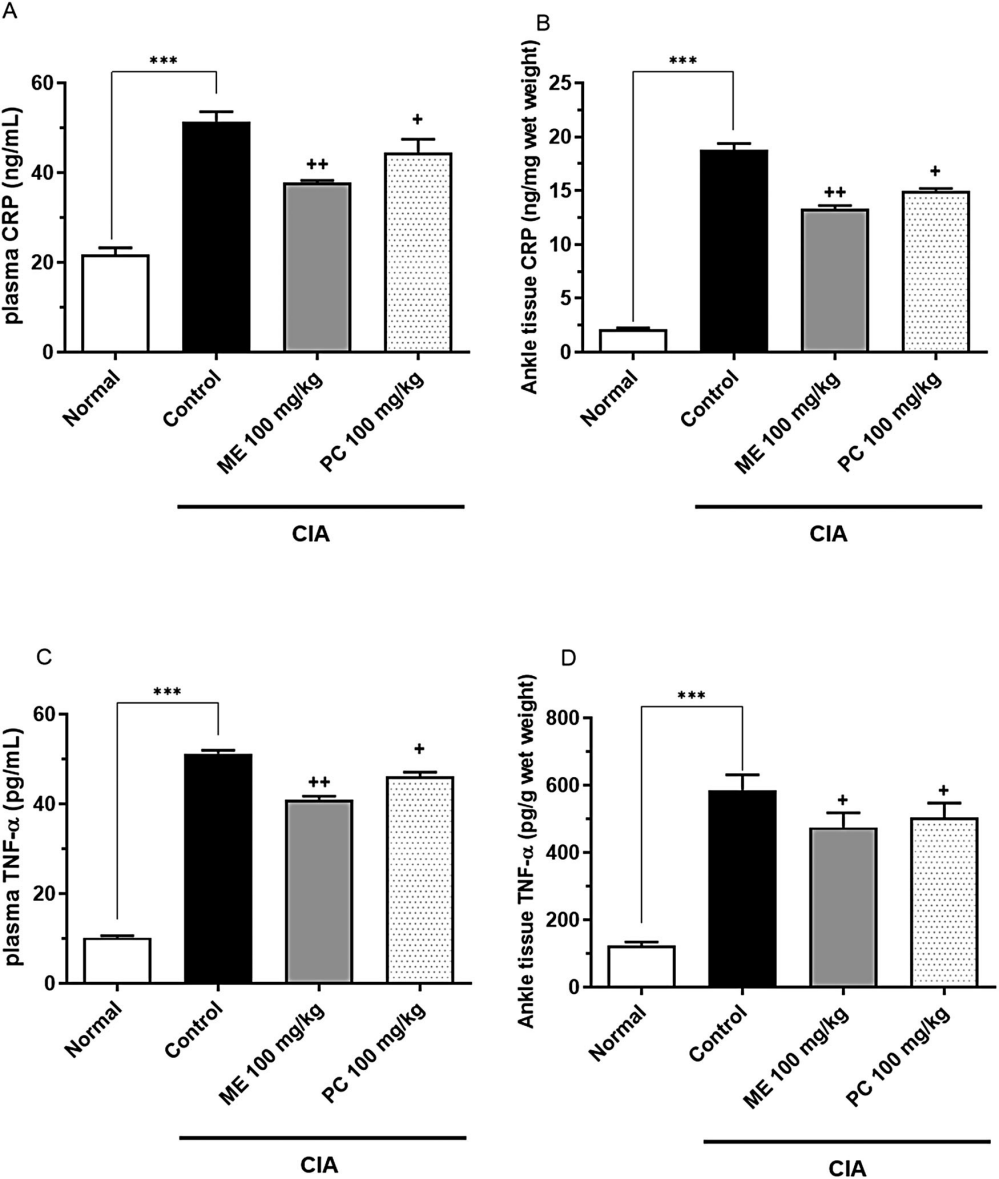
Effect of mixture extract (ME) and *Perna canaliculus* (PC) on C-reactive protein (CRP) and tumor necrosis factor (TNF)-α levels in the plasma and the ankle tissue in the collagen-induced arthritis (CIA) model. Mice were orally administered ME (100 mg/kg) or PC (100 mg/kg) once daily for 1 week. The plasma CRP (A), ankle tissue CRP (B), plasma TNF-α (C), and ankle tissue TNF-α (D) levels were measured 1 h after the last treatment. Blood samples were collected from the tail vein. The ankle tissue was homogenized and the supernatant was separated from the pellet after centrifugation at 4°C. Both CRP and TNF-α levels in the plasma and supernatant were measured using an ELISA kit. Error bars represent SD. Five animals were used in each group (***p <* *0.01, compared to the vehicle-treated group; ^+^*p *<* *0.05, ^++^*p *<* *0.01, compared to the CIA control group).

Four days after the CIA model was established, 100 mg/kg of ME or PC was orally administered repeatedly (once per day for 1 week). The concentrations of CRP and TNF-α in plasma and ankle tissues were measured using ELISA kits. The levels of both TNF-α and CRP in the plasma and ankle tissue were higher in the CIA model group than in the control group, as shown in Figure 2. Both ME and PC attenuated CRP and TNF-α levels in plasma and ankle tissues in the CIA model. The ME-induced effect was more pronounced than the PC-induced effect (Figure 2).

### Effect of ME and PC on COX-1, COX-2, and NF-κB expression levels in ankle tissue of CIA model

**Figure 3.**
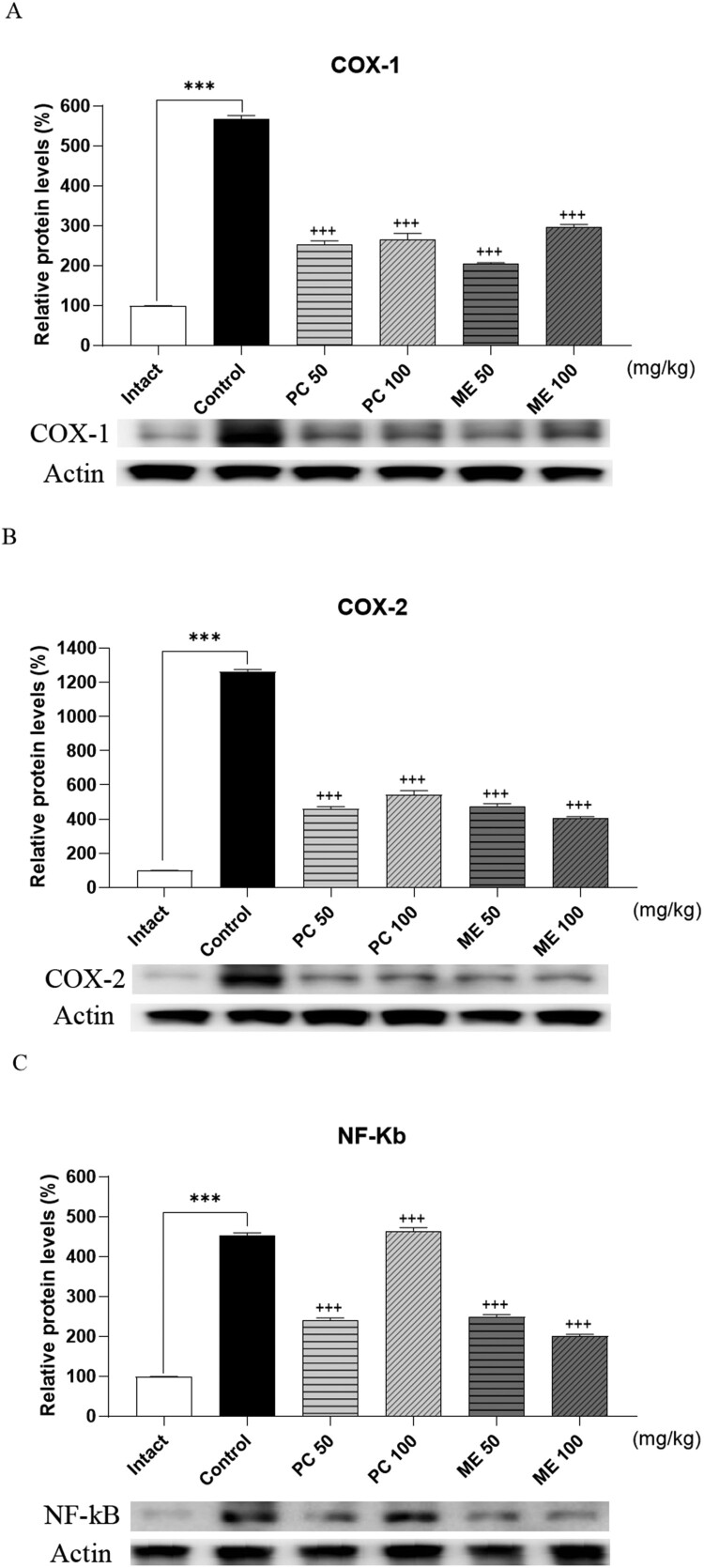
Effect of mixture extract (ME) and *Perna canaliculus* (PC) on COX-1, COX-2, and NF-κB expression in ankle tissue of the collagen-induced arthritis (CIA) model. COX-1, COX-2, and NF-κB levels in the ankle tissue were measured using western blotting after repeated oral administration of 100 mg/kg ME or PC once per day for 1 week. β-Actin was used as an internal control. Values are mean ± SD. These values are expressed as the percentage of COX-1, COX-2, and NF-κB protein/β-actin for each sample (***p <* *0.01, compared to the vehicle-treated group; ^+++^*p *<* *0.01, compared to the CIA control group).

To examine whether ME or PC could cause any change in the expression levels of COX-1, COX-2, and NF-κB, 100 mg/kg of ME or PC was orally administered repeatedly (once per day for 1 week) 4 days after the production of the CIA model, and ankle tissues were dissected for western blotting. As shown in Figure 3, COX-1, COX-2, and NF-κB levels increased in the ankle tissue of CIA mice. Both ME and PC attenuated COX-1, COX-2, and NF-κB expression to almost to the control levels.

### ME and PC down-regulated the expression levels of proinflammatory cytokines levels in ankle tissue of CIA-induced arthritis model

**Figure 4.**
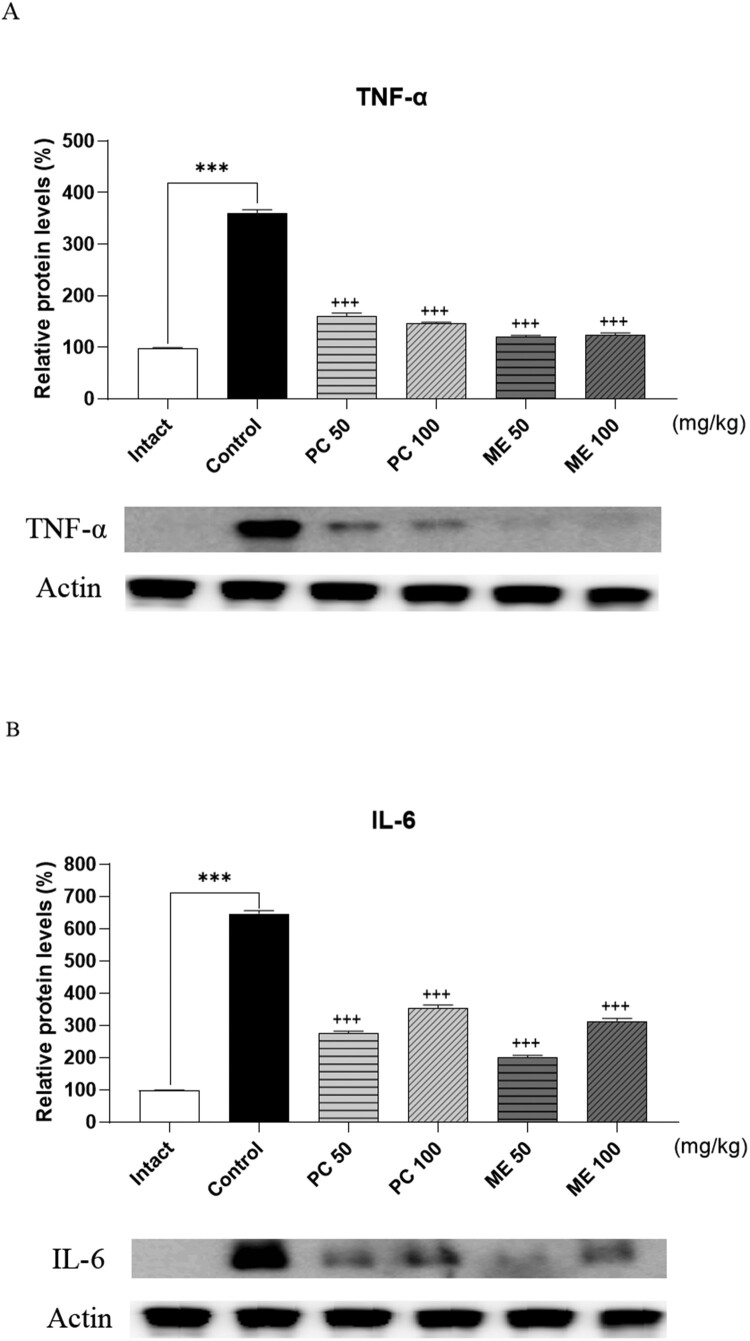
Effect of mixture extract (ME) and *Perna canaliculus* (PC) on tumor necrosis factor (TNF)-α and interleukin (IL)-6 expression in ankle tissue of the collagen-induced arthritis (CIA) model. Mice were orally administered with the vehicle, ME (100 mg/kg), or PC (100 mg/kg) treatments once daily for 1 week. TNF-α and IL-6 protein levels in the ankle tissue were measured using western blotting after repeated oral administration of 100 mg/kg ME or PC once daily for 1 week. β-Actin was used as an internal control. Values are mean ± SD. These values are expressed as the percentage of TNF-α, IL-6 protein/β-actin in each sample (***p <* *0.01, compared to the vehicle-treated group; ^+++^*p *<* *0.01, compared to the CIA control group).

We further examined whether ME or PC could cause any change in the expression levels of proinflammatory cytokines. Four days after the CIA model was established, 100 mg/kg of ME or PC was orally administered repeatedly (once per day for 1 week), and ankle tissues were dissected for western blotting. As shown in Figure 4, TNF-α and IL-6 levels were upregulated in the ankle tissues of CIA mice. Oral pretreatment with ME or PC attenuated the levels of TNF-α and IL-6 expression to almost those of control levels (Figure 4).

## Discussion

In the present study, we found that both short-and long-term treatments with ME produced an antinociceptive effect in a mouse CIA model. The present findings are partly in line with our previous results. First, we previously found that the extracts isolated from AP or SM and the MEs of AP and SM in the MSU-induced gout arthritis model (Hwang et al. 2018). Additionally, we found that a mixture of AP and SM administered orally once was effective for producing antinociception in a mouse MIA model (Feng et al. 2021). Furthermore, repeated administration of ME for 7 days (once daily) made a profound antinociceptive effect, suggesting that antinociceptive tolerance is not induced by ME in the MIA model (Feng et al. 2021). We also discovered in the present study that repeated administration of ME for 7 days (once daily) produced a profound antinociceptive effect in the CIA model.

Several lines of evidence have demonstrated that PC effectively relieves inflammation and arthritis conditions (Lee et al. 2008, 2009; Eason et al. 2019). In addition, PC can reduce inflammation in arthritic patients (Coulson et al. 2015; Eason et al. 2019). Previous studies have shown that PC inhibits the release of some proinflammatory mediators, such as IL-1, IL-2, IL-6, and TNF-α (Lawson et al. 2007; Lee et al. 2008, 2009). In the present study, we found that CRP and TNF-α levels in the plasma and ankle tissues increased in the CIA model and reduced to the control levels with a statistically certainty by ME and PE pre-treatment (50 and 100 mg/kg). This finding is partly similar to our previous study where we observed reductions in CRP and TNF-α production in the plasma of ME- or PC-treated groups in the CIA model. Furthermore, we found in the present study that COX-1, COX-2, NF-κB, TNF-α and IL-6 expression was also reduced in ME- or PC-treated groups (from 50 mg/kg) in the CIA model, suggesting that ME or PC produces an antinociceptive effect by inhibiting the levels of COX-1, COX-2, NF-κB, TNF-α and IL-6 in the CIA model.

When we compared the effects of ME and PC, ME displayed a slightly better antinociceptive and anti-inflammatory effect than PC. Notably, at the same dose of ME and PC, ME showed an earlier onset time for the production of antinociception than PC in the CIA model. The reason that ME appeared more effectively in antinociceptive and anti-inflammatory action than PC in the CIA model was that the decrease in the expression of COX-1, COX-2, NF-Kb, TNF-a, and IL-6 was more effective in ME than PC. Thus, we suggest that ME could be considered a substitute for PC as it is convenient to obtain and is more efficient.

Our findings suggest that ME has the potential to prevent CIA owing to its antinociceptive and anti-inflammatory properties. Additionally, the molecular mechanism of the antinociceptive and anti-inflammatory effects of ME may involve the inhibition of pro-inflammatory cytokine levels in the plasma, such as TNF-α and the suppression of inflammatory mediators such as COX-1, COX-2, NF-κB, TNF-α and IL-6 in the ankle tissue. Taken together, ME could be considered a new potential source for the treatment of arthritis.

## References

[CIT0001] Abramson SB. 2008. Osteoarthritis and nitric oxide. Osteoarthr Cartil. 16:S15–S20. 10.1016/S1063-4584(08)60008-4 18794013

[CIT0002] Ahn HJ, Roh JS, Lee SG, Beon JY, Lee BG, Sohn DH, Kim SY. 2021. Myeloid IPMK promotes the resolution of serum transfer-induced arthritis in mice. Animal Cells Syst. 25(4):219–226. 10.1080/19768354.2021.1952305 PMC8366620 34408810

[CIT0003] Bijlsma JW, Berenbaum F, Lafeber FP. 2011. Osteoarthritis: an update with relevance for clinical practice. Lancet. 377(9783):2115–2126. 21684382 10.1016/S0140-6736(11)60243-2

[CIT0004] Bonin RP, Bories C, De Koninck Y. 2014. A simplified up-down method (SUDO) for measuring mechanical nociception in rodents using von Frey filaments. Mol Pain. 10(1):26. 24739328 10.1186/1744-8069-10-26 PMC4020614

[CIT0005] Chen L, Teng H, Fang T, Xiao J. 2016. Agrimonolide from *Agrimonia pilosa* suppresses inflammatory responses through down-regulation of COX-2/iNOS and inactivation of NF-κB in lipopolysaccharide-stimulated macrophages. Phytomedicine. 23(8):846–855. 27288920 10.1016/j.phymed.2016.03.016

[CIT0006] Choi HG, Tran PT, Lee JH, Min BS, Kim JA. 2018. Anti-inflammatory activity of caffeic acid derivatives isolated from the roots of *Salvia miltiorrhiza* Bunge. Arch Pharm Res. 41(1):64–70. 29124660 10.1007/s12272-017-0983-1

[CIT0007] Choy EH, Panayi GS. 2001. Cytokine pathways and joint inflammation in rheumatoid arthritis. N Engl J Med. 344(12):907–916. 11259725 10.1056/NEJM200103223441207

[CIT0008] Coulson S, Palacios T, Vitetta L. 2015. *Perna canaliculus* (green-lipped mussel): bioactive components and therapeutic evaluation for chronic health conditions. Prog Drug Res. 70:91–132. 26462365 10.1007/978-3-0348-0927-6_3

[CIT0009] Eason CT, Adams SL, Puddick J, Romanazzi D, Miller MR, King N, Johns S, Forbes-Blom E, Feng JH, Lee HJ, et al. 2019. Antinociceptive effect of single components isolated from *Agrimonia pilosa* Ledeb. Extract Sci Pharma. 87(3):18.

[CIT0010] Feng JH, Kim HY, Sim SM, Zuo CL, Jung JS, Hwang SH, Kwak YG, Kim MJ, Jo JH, Kim SC, et al. 2021. The anti-inflammatory and the antinociceptive effects of mixed *Agrimonia pilosa* Ledeb. and *Salvia miltiorrhiza* Bunge extract. Plants. 10(6):1234. 34204404 10.3390/plants10061234 PMC8234973

[CIT0011] Gao H, Sun W, Zhao J, Wu X, Lu JJ, Chen X, Xu QM, Khan IA, Yang S. 2016. Tanshinones and diethyl blechnics with anti-inflammatory and anti-cancer activities from *Salvia miltiorrhiza* Bunge (Danshen). Sci Rep. 6:33720. 27666387 10.1038/srep33720 PMC5036060

[CIT0012] Goldring MB, Goldring SR. 2007. Osteoarthritis. J Cell Physiol. 213(3):626–634. 17786965 10.1002/jcp.21258

[CIT0013] Goldring MB, Otero M. 2011. Inflammation in osteoarthritis. Curr Opin Rheumatol. 23(5):471. 21788902 10.1097/BOR.0b013e328349c2b1 PMC3937875

[CIT0014] Hong JS, Feng JH, Park JS, Lee HJ, Lee JY, Lim SS, Suh HW. 2020. Antinociceptive effect of chrysin in diabetic neuropathy and formalin-induced pain models. Animal Cells Syst. 24(3):143–150. 10.1080/19768354.2020.1765019 PMC7651853 33209194

[CIT0015] Hwang SH, Kim SB, Jang SP, Wang Z, Suh HW, Lim SS. 2018. Anti-nociceptive effect and standardization from mixture of *Agrimonia pilosa* Ledeb and *Salvia miltiorrhiza* Bunge extracts. J Med Food. 21(6):596–604. 29847228 10.1089/jmf.2017.4077

[CIT0016] Jang HH, Nam SY, Kim MJ, Kim JB, Choi JS, Kim HR, Lee YM. 2017. *Agrimonia pilosa* Ledeb. aqueous extract improves impaired glucose tolerance in high-fat diet-fed rats by decreasing the inflammatory response. BMC Complement Altern Med. 17(1):442. 28870184 10.1186/s12906-017-1949-z PMC5583762

[CIT0017] Jeon SJ, Son KH, Kim YS, Choi YH, Kim HP. 2008. Inhibition of prostaglandin and nitric oxide production in lipopolysaccharide-treated RAW 264.7 cells by tanshinones from the roots of *Salvia miltiorrhiza* bunge. Arch Pharm Res. 31(6):758. 18563358 10.1007/s12272-001-1223-4

[CIT0018] Kim CY, Yu QM, Kong HJ, Lee JY, Yang KM, Seo JS. 2020. Antioxidant and anti-inflammatory activities of *Agrimonia pilosa* Ledeb. extract. Evid Based Complement Alternat Med. 2020(8571207):1–10. 10.1155/2020/8571207 PMC7315271 32617113

[CIT0019] Lawson BR, Belkowski SM, Whitesides JF, Davis P, Lawson JW. 2007. Immunomodulation of murine collagen-induced arthritis by N, N-dimethylglycine and a preparation of *Perna canaliculus*. BMC Complem Altern Med. 7(1):1–9. 10.1186/1472-6882-7-20 PMC1899520 17562016

[CIT0020] Lee CH, Butt YKC, Wong MS, Lo SCL. 2008. A lipid extract of *Perna canaliculus* affects the expression of pro-inflammatory cytokines in a rat adjuvant-induced arthritis model. Eur Ann Allergy Clin Immunol. 40(4):148. 19227651

[CIT0021] Lee CH, Lum JHK, Ng CKC, McKay J, Butt YKC, Wong MS, Lo SCL. 2009. Pain controlling and cytokine-regulating effects of lyprinol, a lipid extract of *Perna canaliculus*, in a rat adjuvant-induced arthritis model. Evid Based Complem Alternat Med. 6(2):239–245. 10.1093/ecam/nem100 PMC2686621 18955235

[CIT0022] Libby P. 2008. Role of inflammation in atherosclerosis associated with rheumatoid arthritis. Am J Med. 121(10):S21–S31. 18926166 10.1016/j.amjmed.2008.06.014

[CIT0023] Luo J, Zhang L, Zhang X, Long Y, Zou F, Yan C, Zou W. 2019. Protective effects and active ingredients of *Salvia miltiorrhiza* Bunge extracts on airway responsiveness, inflammation and remodeling in mice with ovalbumin-induced allergic asthma. Phytomedicine. 52:168–177. 30599896 10.1016/j.phymed.2018.09.170

[CIT0024] Mannelli LDC, Piccolo M, Maione F, Ferraro MG, Irace C, De Feo V, Ghelardini C, Mascolo N. 2018. Tanshinones from *Salvia miltiorrhiza* Bunge revert chemotherapy-induced neuropathic pain and reduce glioblastoma cells malignancy. Biomed Pharmacother. 105:1042–1049. 30021339 10.1016/j.biopha.2018.06.047

[CIT0025] McInnes IB, Schett G. 2007. Cytokines in the pathogenesis of rheumatoid arthritis. Nature Rev Immunol. 7(6):429–442. 17525752 10.1038/nri2094

[CIT0026] Ngo TM, Tran PT, Hoang LS, Lee JH, Min BS, Kim JA. 2021. Diterpenoids isolated from the root of *Salvia miltiorrhiza* and their anti-inflammatory activity. Natural Product Res. 35(5):726–732. 10.1080/14786419.2019.1596098 30961363

[CIT0027] Park SH, Sim YB, Kang YJ, Lee JK, Lim SS, Suh HW. 2012. Effect of *Agrimonia pilosa* Ledeb extract on the antinociception and mechanisms in mouse. Korean J Physiol Pharmacol. 16(2):119–123. 22563257 10.4196/kjpp.2012.16.2.119 PMC3339287

[CIT0028] Siriarchavatana P, Kruger MC, Miller MR, Tian HS, Wolber FM. 2019. The preventive effects of greenshell mussel (*Perna canaliculus*) on early-stage metabolic osteoarthritis in rats with diet-induced obesity. Nutrients. 11(7):1601. 10.3390/nu11071601 PMC6683089 31311115

[CIT0029] Wieland HA, Michaelis M, Kirschbaum BJ, Rudolphi KA. 2005. Osteoarthritis-an untreatable disease? Nat.Rev Drug Discov. 4(4):331–344. 15803196 10.1038/nrd1693

[CIT0030] Zhang W, Suo M, Yu G, Zhang M. 2019. Antinociceptive and anti-inflammatory effects of cryptotanshinone through PI3K/Akt signaling pathway in a rat model of neuropathic pain. Chem Biol Interact. 305:127–133. 30922766 10.1016/j.cbi.2019.03.016

